# *Aedes (Stegomyia) aegypti* mosquito bite hypersensitivity in a dog: a case report

**DOI:** 10.1186/s12917-020-02622-x

**Published:** 2020-10-23

**Authors:** Djamel Tahir, Leon Nicolaas Meyer, Nouha Lekouch, Marie Varloud

**Affiliations:** 1 Clinvet Morocco, B.P 301, 28815 Mohammedia, Morocco; 2 Ceva Santé Animale, 10 Avenue de la Ballastière, 33500 Libourne, France

**Keywords:** Case report, *Aedes aegypti*, Dog, Hypersensitivity, Protective measures

## Abstract

**Background:**

Mosquitoes are vectors of several pathogens of considerable importance to humans and companion animals, including nematode helminths such as *Dirofilaria immitis* and *Dirofilaria repens* that cause heartworm disease and subcutaneous dirofilariosis, respectively. In addition to mosquito-borne pathogen transmission, mosquito bites can cause discomfort and irritation in pets, and even lead to severe hypersensitivity reactions. In the present study, we report an acute local hypersensitivity reaction in a dog following experimental exposure to *Aedes* (*Stegomyia*) *aegypti*.

**Case presentation:**

A healthy six-year-old male beagle was included in an efficacy study in which dogs (*n* = 28) were exposed to *Ae. aegypti* mosquitoes. On Day − 6, the dog was allocated to one of the study groups, consisting of seven dogs to be treated on Day 0 with an imidacloprid/flumethrin collar. After sedation, animals were exposed to approximately 50 females of *Ae. aegypti* for 60 (± 5) minutes on Days − 6, 1, 7, 14, 21, 28, 55, and 83. On Day − 6, no allergic reaction to the mosquito bites was observed. However, on Day 1, corresponding to the second challenge, the dog demonstrated an acute allergic reaction characterized by swelling of the face (especially in the base of the muzzle and around the eyes), redness of the eyes, and conjunctival edema of the right eye was also observed. The dog was immediately treated with an intramuscular injection of a commercially available antihistamine treatment, Pen-Hista-Strep® containing a suspension of benzylpenicillin, chlorphenamine, dexamethasone, dihydrostreptomycin, and procaine at a dosage of 1 mL per 10 kg. A few hours after treatment, the dog showed noticeable improvement.

**Conclusions:**

This case provides the first evidence of canine acute local hypersensitivity reaction to mosquito bites under laboratory conditions. This observation suggests that invasive mosquito species such as *Aedes* spp. may affect the health and comfort of our companion animals, especially for pets with outdoor access without individual protective measures against insect bites.

## Background

Companion animals can be bitten by numerous blood-sucking ectoparasites such as ticks, fleas, sand flies, and mosquitoes. Ectoparasites directly impact animal health through bites (e.g., irritation, pain, nodular reactions, and hypersensitivity reactions) and indirectly by their ability to transmit pathogens (e.g., *Acanthocheilonema reconditum*, *Dipylidium caninum*, *Bartonella* spp., *Leishmania infantum*, and *Dirofilaria immitis*) [1].

An acute allergic reaction is an immediate hypersensitivity reaction to a foreign substance, often known as an allergen or antigen [2]. In dogs, the pathophysiology of the acute allergic reaction may be divided into two steps: sensitization and provocation. The first step starts following the first contact with an allergen when it enters through the skin, during which a short-term localized reaction called a humoral response occurs [3]. This response stimulates B cells to switch to immunoglobulin E (IgE) production. The IgE produced by these local B cells binds tissue mast cells, which are subsequently sensitized and can respond by degranulation and prostaglandin and leukotriene synthesis, inducing the symptoms of an allergic reaction (redness and swelling at the bite site). This entire process can take weeks, months, or even years to develop [4]. During the provocation step, sensitized mast cells recognize allergens and release their contents in a process known as degranulation and activation. A localized allergic reaction, such as extreme swelling at the bite site, will occur. In most cases, no hypersensitivity appears, and the individual does not become allergic because the immune system regulates itself to produce more IgG than IgE. In severe reactions, the localized reaction causes further release of the contents of mast cells throughout the body, resulting in systemic anaphylaxis [3].

When a dog presents with signs of a hypersensitivity reaction, there is often no evidence to clearly indicate the source. In contrast to fleas and ticks, mosquitoes feed rapidly and leave their hosts within 3–5 min. This behavior may explain why severe reactions to mosquito bites have been poorly documented in dogs. In the present report, we describe mosquito bite hypersensitivity (BH) observed in a dog after experimental exposure to *Ae. aegypti*.

## Case presentation

The present case was observed following an efficacy study conducted using four groups of seven dogs each. All dogs were from Clinvet colony, bred at the facility. On Day − 6, animals were sedated by intramuscular injection of medetomidine (Domitor®, 100 µg/kg; equivalent of 0.1 mL/kg) and then exposed individually to approximately 50 unfed females of *Ae. aegypti* for 60 (± 5) minutes. For the dog mentioned in this case report, 46 live blood-fed mosquitoes were recorded at the end of the challenge. After this assessment, the dog (six-year-old male beagle, weighing 12 kg) was enrolled with six other animals in one of the treated groups. Each dog in this group was fitted with an imidacloprid/flumethrin collar on Day 0 and then observed for any adverse effect attributable to product treatment. All dogs were exposed individually to mosquitoes (*N* = 50) on Days 1, 7, 14, 21, 28, 55, and 83. Clinical examinations were performed 1–2 hours before each exposure of dogs to mosquito bites. It is particularly noteworthy that the dog had not been previously exposed to *Ae. aegypti* at the facility. Furthermore, all dogs are not exposed to wild mosquitoes that might exist in the area where the facility is implemented.

**Fig. 1:**
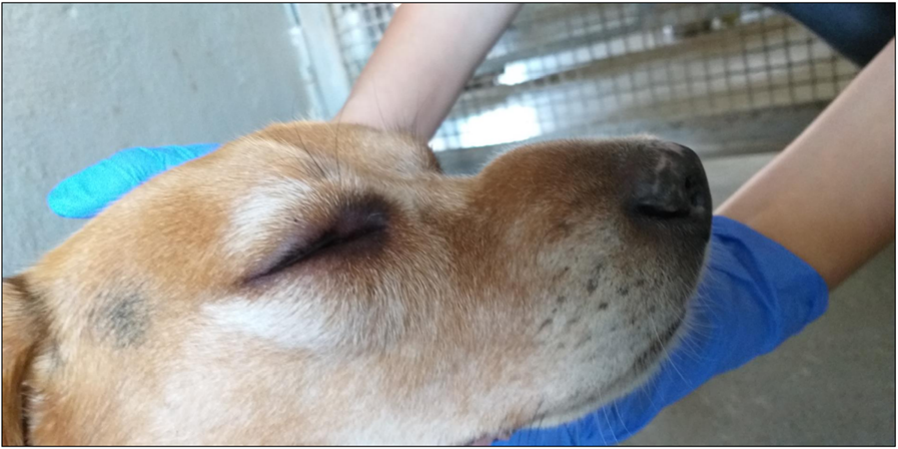
Swelling on the face including the muzzle and around the eyes of dog exposed to *Aedes* mosquitoes

**Fig. 2:**
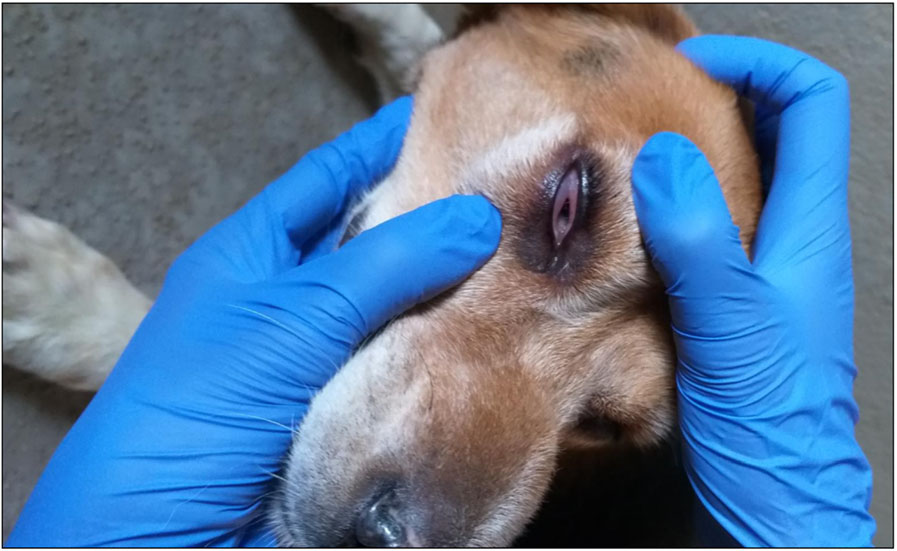
Redness and conjunctival edema of the right eye of dog exposed to *Aedes* mosquitoes

On Day 1, when dogs were challenged with mosquitoes, one dog presented an acute allergic reaction to mosquito bites within 40 min after exposure. This reaction was characterized by swelling of the face, especially in the base of the muzzle and around the eyes (Fig. 1), a redness of the eyes, and conjunctival edema of the right eye (Fig. 2). The rectal temperature was 38.4 °C. To avoid progression of the reaction into potentially fatal anaphylactic shock, the dog was removed from the study and immediately treated with an intramuscular injection of an antihistamine suspension containing benzylpenicillin, chlorphenamine, dexamethasone, dihydrostreptomycin, and procaine (Pen-Hista-Strep®) at 1 mL/10 kg body weight. The dog was observed for adverse effects for 3 hours. Live fed mosquitoes were collected from the dog during this second challenge. A few hours after treatment, the dog improved clinically. No hypersensitivity reactions were observed in the other dogs throughout the study (*N* = 27).

## Discussion and conclusions

To the best of our knowledge, there are no formal published data concerning allergic responses of dogs following mosquito bites. Pets may have intense allergies to tick and flea bites. Clinical signs include red, dry, or irritated skin; excessive itching or scratching and dermatitis; as well as lethargy, anorexia, and high fever [5]. The present report describes the clinical evolution of the acute local hypersensitivity reaction and provides new information regarding clinical signs in a dog after experimental exposure to mosquito bites. In comparison, the most common symptoms observed in cats manifesting cutaneous reactivity to mosquito bites are erythematous papules on the ear pinnae, and ulcerative and crusted dermatitis of the face, ears, and paws [6, 7].

Though only nine mosquitoes had successfully taken a blood meal from the dog during the Day 1 challenge, this number was enough to trigger an acute allergic reaction. Apparently, the dog was strongly sensitized to mosquito bites during the first massive exposure (46 fully engorged mosquitoes were collected at the end the exposure), during which anticoagulants and other proteins in the saliva of the mosquito were injected into the skin by the feeding mosquitoes. This high dose of allergen received by the dog during the single hour of exposure may be the trigger factor allowing the dog’s immune system to quickly produce high levels of IgE. Hence, the plasmatic IgE threshold may have been reached during the one week preceding the second challenge. In most cases, the immune system regulates itself so that more IgG than IgE is produced, meaning no hypersensitivity appears [3]. Genetic factors are essential for the levels of circulating IgE [3], and may explain why the remaining dogs living in the same environmental conditions and subjected to the same experimental situations did not show any sign of hypersensitivity (*n* = 27).

For the present case, during the second challenge corresponding to one week after initial exposure, the reacting dog may have had a high concentration of circulating specific IgE antibodies against *Ae. aegypti* mosquito saliva. This could explain the rapid allergic reaction (less than one hour) observed. An early IgE response may be observed as early as three to four days following antigen/allergen contact [3]. Furthermore, the occurrence of localized cutaneous reactions to mosquito bites is related to IgE-mediated immediate (75%) or delayed‐type reactions (50%) [6]. According to Galli et al. [8], when the immune system is stimulated by specific allergens, the mastocyte (mast cells) present in the tissue release histamine and heparin. These molecules have an inflammatory effect on the tissues, resulting in the itchy and inflamed skin conditions characteristic of most allergic reactions in canines.

In summary, this observation suggests that mosquitoes may not only play a role as a vector of pathogens for pets, but may also induce serious discomfort during their blood meals in which they inject allergens serving as agents that trigger an acute local hypersensitivity reaction.

## Data Availability

All data used in the current study are available from the corresponding author on reasonable request.

## Abbreviations

BH: Bite hypersensitivity
Ig: Immunoglobulin
